# Large-Scale Microbiome Profiling of Brown Algae Identifies a Specific *Vibrio aphrogenes* Clade Strain That Promotes Gametophyte Settlement

**DOI:** 10.4014/jmb.2604.04061

**Published:** 2026-06-01

**Authors:** Yurim Bae, Dong Jin Kim, Yerin Seo, Yeonjin Noh, Sujin Lee, Soon-Kyeong Kwon

**Affiliations:** 1 Division of Applied Life Science (Brain Korea 21), Gyeongsang National University, Jinju 52828, Republic of Korea; 2 Department of Biological Science, Chosun University, Gwangju 61452, Republic of Korea

**Keywords:** Brown algae, Phycosphere, Gametophyte settlement, *Vibrio aphrogenes*, Biofilm, Algae-microbe interaction

## Abstract

Marine macroalgae are foundation species of coastal ecosystems, maintaining close interactions with their microbiome for development and environmental adaptation. Although secure attachment to substrates is essential for both morphological development and survival, the potential causal link between microbial symbionts and this fundamental attachment process remains poorly understood. To address this gap, we integrated large-scale field sampling with functional validation to identify bacteria that influence the attachment of brown algae, specifically comparing attached and drifted individuals of *Undaria pinnatifida* and *Ecklonia cava*. Notably, *Vibrio* was consistently enriched in attached algae, whereas *Marinomonas* dominated drifted individuals. Translating these field observations into lab-scale validation, we isolated and evaluated their biofilm-formation capacity and impact on gametophyte settlement. A specific isolate belonging to the *Vibrio aphrogenes* clade exhibited superior biofilm formation and significantly enhanced algal abundance 2.57-fold in co-culture with *U. pinnatifida* gametophytes. These findings provide a scientific basis for developing bacterial inocula for seaweed seedling production and support broader macroalgae restoration strategies under changing environmental conditions.

## Introduction

Marine macroalgae are key primary producers in coastal ecosystems and play crucial roles in global nutrient cycling and carbon sequestration [1, 2]. Recent studies have increasingly recognized that macroalgae exist as holobionts, in which associated microbial communities influence host physiology, development, and environmental adaptation [3, 4]. In macroalgal systems, both mutualistic and antagonistic interactions occur depending on microbial species and environmental context [5, 6]. For example, symbiotic bacteria can enhance algal productivity by supplying essential metabolites such as phytohormones, B vitamins, siderophores, or other micronutrients [4, 5, 7, 8]. In addition, bacterial metabolism of algal-derived compounds such as dimethylsulfoniopropionate (DMSP) can contribute to sulfur cycling and influence host stress responses [9, 10]. Conversely, some bacteria compete with algae for dissolved organic carbon and nutrients, produce algicidal compounds, or induce disease [6, 11, 12].

Brown algae (Phaeophyceae), including *Ecklonia cava* and *Undaria pinnatifida*, are major components of underwater forests and play essential roles in coastal ecosystems [13]. Brown algae exhibit a typical Laminariales life cycle, with alternating gametophytic and sporophytic generations. In the coastal waters of South Korea, sporophytes emerge in October and November and grow rapidly from winter to spring when sea temperatures range from 10 to 16°C [14, 15]. In particular, *U. pinnatifida* is a major aquaculture species in Korea, with production reaching approximately 572,000 tons in 2024 and an average annual production of around 548,000 tons in recent years [16]. In addition, *E. cava* has been widely studied as a rich source of phlorotannins exhibiting diverse bioactivities, including anti-cancer, anti-inflammatory, and anti-diabetic effects [17-20]. These characteristics highlight the ecological and economic importance of brown algae in coastal ecosystems.

Interactions between microbes and algae have been extensively studied in marine systems such as coral symbioses, diatoms, and dinoflagellates. However, microbial communities associated with macroalgae [21-24], including brown algae, remain comparatively understudied. In brown algae, early developmental stages involve the establishment of polarity in fertilized zygotes, leading to the formation of basal structures for attachment and subsequent development into macroscopic sporophytes [25]. Given the importance of this settlement process, microbial communities associated with algal and substrate surfaces are likely to play a critical role in mediating these interactions.

Microorganisms rapidly colonize available substrates on marine surfaces, where they engage in diverse ecological interactions. These microbes can influence host development through multiple mechanisms, including chemical signaling, metabolic exchanges, and the modulation of nutrient availability. In algae, such interactions have been implicated in regulating host morphogenesis and growth [26-29]. However, the specific mechanisms by which microbial communities influence early developmental processes in brown algae remain poorly understood.

In this study, we hypothesized that the microbiomes associated with brown algae differ systematically between naturally attached and drifted individuals, and that specific attachment-enriched bacteria play a role in promoting early-stage algal development. To verify this hypothesis, we analyzed the microbiomes of two major brown algae, *E. cava* and *U. pinnatifida*, across the Korean coast. We compared the bacterial communities of attached and drifted algal samples to identify microbial taxa potentially involved in algal attachment. Furthermore, we isolated representative bacterial strains to evaluate their biofilm-forming capacity and conducted co-culture experiments with *U. pinnatifida* female gametophytes to assess their functional impacts. Our findings provide insights into the functional roles of macroalgal-associated microbiota during early developmental stages and highlight the potential importance of biofilm-mediated interactions in maintaining macroalgal populations under changing environmental conditions.

## Materials and Methods

### Sample Collection

Brown algal tissues were collected from December 2021 to September 2022 across 16 sampling events along the coasts of the South Sea, West Sea, East Sea, and Jeju Island, South Korea. A total of 123 individuals of *E. cava* and 222 individuals of *U. pinnatifida* were collected. In the West Sea, where *E. cava* is not naturally distributed, only *U. pinnatifida* was collected. Sampling was conducted at a depth of approximately 1 m using SCUBA diving. Immediately after collection, holdfasts and thallus were separated and placed into sterile 50 mL conical tubes. Samples were transported to the laboratory on ice and stored at -80°C until DNA extraction. For each sampling event, 2 L of surrounding seawater was collected as a control and filtered through 0.22 μm Sterivex filters (Merck Millipore, Germany), with the exception of the Jongdal site (sampling date: 2022-04-16; coordinates: 33.4968666, 126.910263). Sterivex filters were preserved in DNA/RNA Shield (Zymo Research, USA) and stored at -80°C until processing. Environmental parameters including time, date, latitude, longitude, salinity, dissolved oxygen (DO), pH, and temperature were recorded at each site. In addition, biological characteristics of each sample, including species, tissue type, attachment status, life stage, and thallus length were documented.

### DNA Extraction and Sequencing Processing

Total genomic DNA was extracted to analyze microbiome composition using amplicon sequencing. For algal samples, approximately 1 g of thallus and holdfast tissues were collected using sterile scissors and forceps and transferred to homogenizing tubes. DNA was extracted using the Quick-DNA Plant/Seed Miniprep Kit (Zymo Research) according to the manufacturer’s instructions. For seawater samples, microbial biomass collected on 0.22 μm filters was cut into small pieces and transferred to homogenizing tubes, and DNA was extracted using the same kit as described above. The V3–V4 region of the bacterial 16S rRNA gene was amplified using universal primers 341F (5’-CCT ACG GGN GGC WGC AG-3’) and 785R (5’-GAC TAC HVG GGT ATC TAA TCC-3’). Amplicon libraries were prepared using the TruSeq Nano DNA Library Preparation Kit (Illumina, USA) and sequenced on the NovaSeq 6000 platform (Illumina).

### Analysis of Brown Algae-Associated Microbiome

Raw 16S rRNA gene amplicon sequences were processed using QIIME 2 [30]. Paired-end reads were denoised and merged using DADA2 [31] to generate amplicon sequence variants (ASVs). Taxonomic assignment was performed against the SILVA database [32, 33]. Non-bacterial sequences and low-abundance features (< 0.01% of total sequences) were removed. Microbial community structures were analyzed based on Bray–Curtis dissimilarity and weighted and unweighted UniFrac distances, and visualized using principal coordinates analysis (PCoA). Alpha diversity differences between groups were evaluated using the Kruskal–Wallis test with pairwise comparisons. Differences in community composition among coastal regions and attachment status were evaluated using permutational multivariate analysis of variance (PERMANOVA).

Pairwise differential abundance analysis of bacterial genera was performed to identify taxa differing between ecological states (Attach, Drift, and Seawater). Relative abundances were compared using the Wilcoxon rank-sum test, and statistical significance was assessed based on raw *p*-values (*p* < 0.05). Log2 fold changes (log2FC) were calculated based on mean relative abundances between groups with the addition of a pseudocount (1 × 10^−6^) to avoid division by zero.

Functional profiles of microbial communities were predicted from 16S rRNA gene sequences using PICRUSt2 [34], and module-level differences were further analyzed. To identify the key biological features discriminating between the ecological states, Random Forest [35] supervised classification was performed on both microbial taxonomic profiles and predicted metabolic pathways. The analysis was implemented using the ‘randomForest’ package (v4.7-1.2) in R (v4.5.3). The dataset was randomly partitioned into a training set (70%) and a test set (30%) with a fixed seed (123) for reproducibility. The model was constructed using 500 trees (n_tree_ = 500), and its predictive performance was evaluated by applying the trained model to the test set and generating a confusion matrix to determine classification accuracy. The relative importance of individual taxa and functional modules was quantified using the Mean Decrease in Accuracy score (MDA). ROC curves were generated for the top-ranked taxa and functional modules identified by Random Forest, and the area under the curve (AUC) was calculated to assess their classification performance. Model performance was evaluated using out-of-bag (OOB) estimation, an inherent cross-validation procedure in the random forest algorithm.

### Isolation and Identification of Brown Algae-Symbiotic Microbes

Symbiotic bacteria associated with algal attachment status were isolated from brown algal samples. Approximately 1 g of holdfast tissue was transferred into a 50 mL conical tube containing 10 mL of marine broth and ten 3 mm beads. Samples were vortexed for 1 min to detach associated bacteria. The supernatant was serially diluted and spread onto specific culture media to accommodate the growth requirements of different strains: TCBS (thiosulfate–citrate–bile salts–sucrose) agar was used for the selective isolation of *Vibrio* species, while Marine Agar (MA) was employed for the cultivation of *Marinomonas* strains. All plates were subsequently incubated at 25°C under aerobic conditions. For taxonomic identification, colonies were analyzed based on the protocol described by Zhang *et al*. [36]. Specifically, the isolated strains were identified through 16S rRNA gene sequencing to determine their phylogenetic positions and confirm their taxonomic assignments at the species level.

Full-length 16S rRNA gene sequences of *Vibrio* and *Marinomonas* isolates were obtained by PCR amplification using the universal primers 27F and 1492R, followed by sequencing. Taxonomic identification was performed using the EZBioCloud database [37]. Full-length sequences were aligned using MUSCLE [38], and phylogenetic analysis was conducted. We constructed a large-scale tree using FastTree [39, 40] to accommodate a vast number of reference sequences and provide a broad taxonomic context. A subset of closely related sequences was selected for maximum likelihood phylogenetic analysis in MEGA X [41].

### Biofilm Formation Capacity of Isolated Bacterial Strains

Biofilm biomass was quantified via a crystal violet staining assay. A *Marinomonas* strain was utilized as a negative control. Overnight cultures were grown in marine broth at 25°C under aerobic conditions with shaking. Subsequently, 200 μL of each culture was inoculated into 96-well plates and incubated statically at 25°C for 24 h. Cell density was measured at 600 nm using a microplate reader (Infinite^®^ 200 PRO). The culture supernatant was then removed, and the plates were air-dried at room temperature for 1 h. Adherent cells were stained with 0.1% crystal violet for 30 min, followed by washing with distilled water to remove excess stain. The bound dye was solubilized using absolute ethanol, and biofilm biomass was quantified by measuring absorbance at 595 nm. Marine broth without inoculation was used as a medium blank, and all experiments were performed in quadruplicate. Statistical significance was assessed using Student’s t-test. The measured values were normalized using the following equation.

$$\text{Normalized biofilm formation =}\frac{{\text{OD}}_{\text{595}}\text{ nm}\left(\text{bacteria}\right)-{\text{OD}}_{\text{595}}\text{ nm}\left(\text{medium blank}\right)}{{\text{OD}}_{600}\text{ nm}\left(\text{bacteria}\right)-{\text{OD}}_{600}\text{ nm}\left(\text{medium blank}\right)}$$

### Co-Culture Experiments with *U. pinnatifida* Female Gametophytes and Isolated Strains

Co-culture experiments were conducted to evaluate interactions between bacterial isolates and brown algae female gametophytes. The bacterial isolates were cultured in marine broth (MB) at 25°C with shaking at 180 rpm for 24 h. The cultures were adjusted to a similar optical density (OD_600_ = 0.2) prior to use to minimize variation in initial cell density. Subsequently, 1 mL of each culture was transferred into individual wells of sterile 24-well plates and incubated statically at 25°C for an additional 24 h to allow biofilm formation on the well surface. After incubation, the culture supernatant was carefully removed, and wells were gently washed with sterile artificial seawater to remove non-adherent cells.

For algal co-culture, approximately 0.1 g of *U. pinnatifida* female gametophytes were aseptically cut into small fragments using a sterile razor blade and suspended in PES medium. The suspension was filtered through a 0.45 μm membrane to obtain a uniform suspension. The resulting suspension was mixed thoroughly to ensure even distribution of algal material. Subsequently, 1 mL of the algal suspension was added to each well containing preformed bacterial biofilms. Co-cultures were incubated statically at 20°C under a 14:10 h light:dark cycle for 2 days to facilitate initial attachment, followed by incubation under continuous light with shaking at 100 rpm for 10 days to promote growth. After incubation, the supernatant was carefully removed to eliminate non-attached algal cells. The remaining attached algal biomass was collected by scraping the well surface using a sterile cell scraper and transferred to homogenizing tubes. DNA extraction was performed using the Quick-DNA Plant/Seed Miniprep Kit (Zymo Research) according to the manufacturer’s instructions.

Algal abundance was quantified by quantitative PCR (qPCR) targeting the housekeeping gene eukaryotic elongation factor 1 beta (eEF1β, forward primer : 5’-CAG TAG TCA CCG TGG CTA TTG C-3’; reverse primer: 5’- CGG CAA ACG AAA CAA CGG TA-3’). Absolute quantification was performed using a standard curve generated from serial dilutions (slope = −3.971, y-intercept = 29.517, R^2^ = 0.983, efficiency = 78.58%, error = 0.113). Marine broth without bacterial inoculation was used as a blank, and all experiments were performed in quadruplicate. Statistical significance was assessed using Student’s t-test. This assay was designed to quantify the amount of algal biomass retained on pre-formed bacterial biofilms and therefore primarily reflects bacterial effects on algal attachment and persistence rather than direct algal growth.

## Results

### Coastal Region, Host Species, and Attachment Status Jointly Shape Brown Algal Microbiomes

**Fig. 1:**
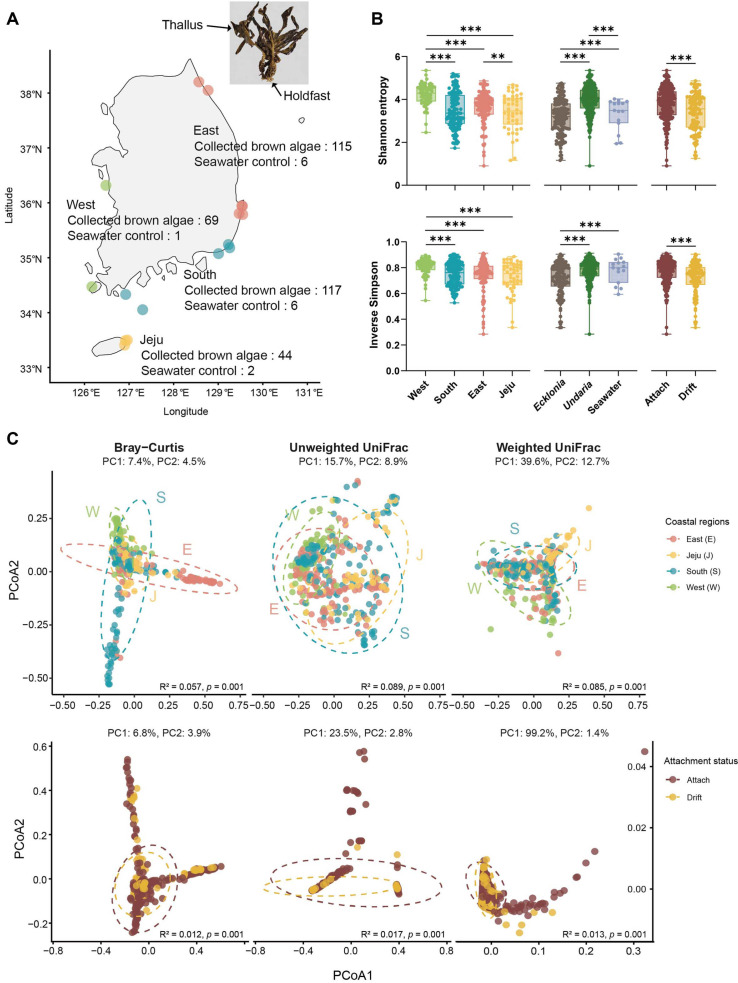
Geographic distribution and diversity patterns of macroalgal-associated microbial communities. (**A**) Map showing sampling locations of brown algae along the eastern, western, southern, and Jeju coasts of South Korea. Samples were collected from 16 distinct coastal sites. Sampling regions are indicated by colored circles (eastern: pink, western: green, southern: blue, Jeju: yellow). (**B**) Comparisons of bacterial alpha diversity across coastal regions, host species, and attachment status. Alpha diversity was assessed using Shannon diversity and evenness indices. Statistical significance was determined using the pairwise Kruskal–Wallis test (***, *p* < 0.001; **, *p* < 0.01; *, *p* < 0.05). (**C**) Beta diversity of microbial communities based on Bray–Curtis dissimilarity and both weighted and unweighted UniFrac distances. Statistical significance was assessed using permutational multivariate analysis of variance (PERMANOVA).

To characterize the microbiota profiling at the phycosphere of the brown algae, we analyzed 345 algal samples from *E. cava* (n = 123) and *U. pinnatifida* (n = 222), together with 15 seawater controls (360 samples in total). These samples were collected from 16 coastal sites across four major regions: the East Sea, West Sea, South Sea, and Jeju Island (Fig. 1A).

To ensure a comprehensive analysis of the algal-associated microbiome, we utilized both holdfast and thallus tissues. Specifically, the *E. cava* dataset comprised 69 holdfast and 54 thallus samples, while the *U. pinnatifida* dataset included 121 holdfast and 101 thallus samples. Samples were categorized by their attachment status into attached (n = 238) and drifted (n = 107) groups to investigate the microbial drivers of algal attachment status (Table S1 and S2).

The datasets for the brown algae groups comprised 2,764 ASVs classified into 901 genera, 505 families, 266 orders, 106 classes, and 47 phyla. Amplicon sequencing analysis revealed that the microbial community was dominated by three major phyla: Proteobacteria (71.7% ± 19.0), Bacteroidetes (9.8% ± 11.4), and Planctomycetes (5.7% ± 11.1). We next examined differences in microbial community composition between seawater and brown algal samples. While Bacteroidetes and Actinobacteria were relatively more abundant in seawater samples (26.8% ± 14.4 and 7.6% ± 10.5, respectively), brown algal samples were characterized by increased relative abundances of Planctomycetes (6.0% ± 11.3) and Verrucomicrobia (4.50% ± 9.2). In addition, several low-abundance phyla, including Firmicutes and Cyanobacteria, were detected exclusively or predominantly in algal samples, suggesting the presence of host-associated microbial niches (Fig. S1). Substantial variation in the relative abundance of dominant phyla was observed across samples indicating heterogeneous community structures among samples. Given these compositional differences, alpha diversity analysis revealed significant differences between seawater and both *E. cava*- and *U. pinnatifida*-associated microbiomes (Fig. 1B). Our dataset exhibited spatial patterns, with samples from the West Sea showing distinct community structures compared to other coastal regions, while additional differences were observed between the East Sea and Jeju Island. These results suggest that geographic variation contributes to differences in microbial community composition across coasts. Microbial diversity varied significantly depending on attachment status. There were significant differences in alpha diversity indices including Shannon index (*p* < 0.05) and Inverse Simpson index (*p* < 0.05) between attachment and drift status. Beta diversity analyses revealed statistically significant but modest compositional differences associated with attachment status across all distance metrics (R^2^ = 0.012~0.017, *p* < 0.05, Fig. 1C). Given the small effect sizes, these results suggest that attachment status contributes to modest ecological differentiation of the brown algal microbiome rather than acting as a dominant driver of overall community structure.

### Microbial Taxa and Functional Modules Associated with Algal Attachment Status

**Fig. 2:**
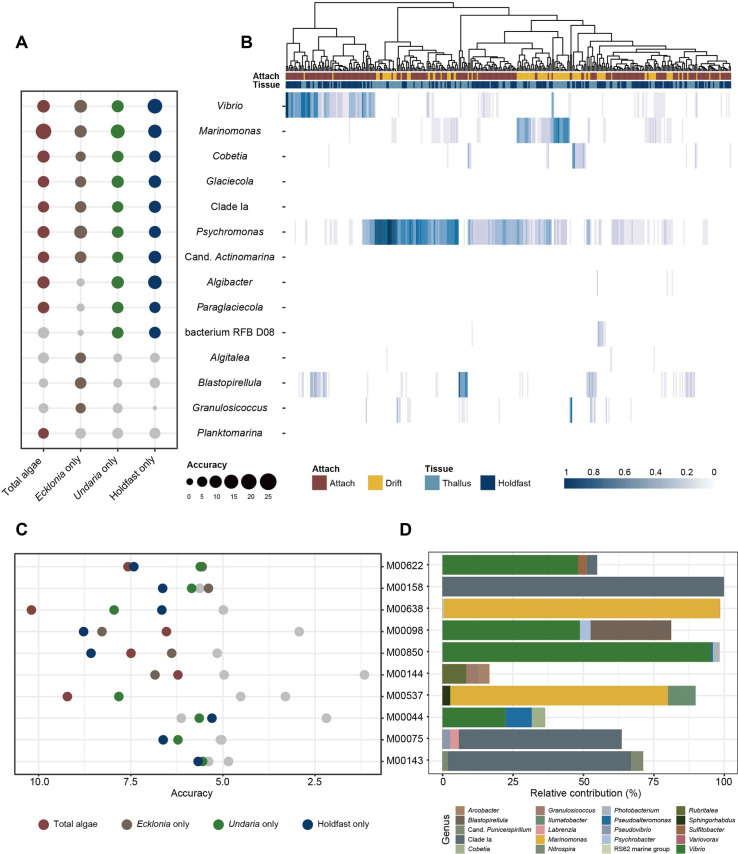
Random Forest analysis identifying key taxa and functional modules associated with attachment status and seawater controls. (**A**) Random Forest classification of microbial communities across four stratified datasets to identify key predictors distinguishing attached and drifting algal samples in comparison with seawater controls. The analysis was performed using (i) all brown algae samples combined, (ii) species-specific datasets (*E. cava* and *U. pinnatifida*), and (iii) tissue-specific samples (holdfast only). Colored circles indicate taxa ranked within the top 10 most important predictors in at least one dataset, whereas grey circles represent taxa not included in the top 10. Circle size reflects the Mean Decrease in Accuracy (MDA), with larger circles indicating greater contribution of each taxon to model classification performance. (**B**) Heatmap showing the relative abundance of taxa. This panel displays the distribution of the key taxa identified in Fig. 2A across all sample groups. (**C**) Predicted functional modules associated with attachment status and seawater controls. Functional profiles were predicted using PICRUSt2 based on 16S rRNA gene sequences and ranked according to Random Forest importance scores. Filled circles indicate modules ranked within the top 10 based on MDA in at least two datasets, whereas grey circles represent modules not included in the top 10. (**D**) Relative contribution of dominant taxa to the predicted functional modules shown in Fig. 2C. Bar plots display the top three microbial taxa contributing to each functional module, highlighting potential taxon-function associations linked to attachment status. M00622, Nicotinate degradation; M00158, F-type ATPase, eukaryotes; M00638, Salicylate degradation; M00098, Acylglycerol degradation; M00850, *Vibrio* cholerae pathogenicity signature; M00144, Tyrosine degradation; M00537, N-glycan biosynthesis; M00044, NADH dehydrogenase (ubiquinone) Fe-S protein/flavoprotein complex, mitochondria; M00075, NADH:quinone oxidoreductase, prokaryotes; M00143, Xylene degradation.

To identify microbiota more tightly associated with brown algae attachment, Random Forest analysis was performed to distinguish bacterial communities between attached and drifted algae. Seawater samples were included as environmental controls to assess whether the identified discriminatory taxa were specifically associated with algal samples rather than simply reflecting their abundance in the surrounding seawater. The models showed generally significant classification performance across comparison conditions for both taxonomic and functional profiles (*p* < 0.05), although the functional module-based classification in *U. pinnatifida* was not statistically significant (*p* = 0.20), indicating weaker discriminatory power in this subset. However, all models exhibited low OOB error rates (< 12.3%), supporting the overall reliability of the microbial classification. Among the bacterial genera, *Vibrio*, *Marinomonas*, *Cobetia*, *Paraglaciecola*, SAR11 clade Ia, *Psychromonas*, and *Candidatus Actinomarina* were consistently identified as key discriminative genera contributing to the separation of attachment states across comparison conditions, regardless of host species or tissue type (Fig. 2A). To further identify core taxa specifically associated with attachment status, an additional Random Forest analysis comparing only attached and drifting samples was performed, in which *Vibrio* and *Marinomonas* were consistently selected across all comparison conditions (Fig. S2A). Their classification performance was supported by ROC curves from random forest models (Fig. S3). These results suggest a robust association with attachment-related microbial differentiation. The relative abundance patterns of these genera further supported their association with attachment status. At the genus level, both attached and drifting brown algal samples were dominated by *Psychromonas*, showing the highest relative abundance in both groups (22.4% ± 21.0 and 32.3% ± 26.7, respectively). However, differences in secondary dominant taxa were observed depending on attachment status. Drifting samples were characterized by higher relative abundances of *Marinomonas* (21.0% ± 19.3) and *Cobetia* (4.7% ± 10.2), whereas attached samples showed enrichment of *Vibrio* (16.3% ± 19.5) (Fig. 2B and Table S3).

Functional profiling inferred from 16S rRNA gene data using PICRUSt2 revealed that attachment-associated microbial communities were enriched in diverse metabolic modules. In particular, the nicotinate degradation module (M00622) was consistently detected across all comparison conditions, while modules related to salicylate degradation (M00638), F-type ATPase (M00158), acylglycerol degradation (M00098), and the *Vibrio* cholerae pathogenicity signature (M00850) were repeatedly identified across multiple conditions (Fig. 2C). Analysis of taxonomic contributions to these functional modules showed that *Vibrio* was among the major contributors, serving as the dominant taxon in four out of ten modules with relative contributions ranging from 22.4% to 95.3% (median = 48.4%). *Marinomonas* and SAR11 clade Ia were also highly represented across multiple attachment-associated modules (Fig. 2D). When the analysis was restricted to attached and drifting samples, the key attachment-associated functional patterns were largely preserved, with *Vibrio* and *Marinomonas* remaining the dominant contributors across multiple modules (Fig. S2B).

These results indicate that attachment-associated microbial communities are not only compositionally distinct but also functionally specialized. Brown algae are known to release dissolved organic matter into the surrounding environment, enriching the phycosphere. This includes a variety of polyphenolic compounds, such as flavonoids, phenolic terpenoids, phlorotannins, and mycosporine-like amino acids [42-44]. The enrichment of modules related to aromatic compound degradation in attached samples suggests an enhanced capacity for the breakdown and utilization of complex algal-derived organic substrates.

### Functional Characterization of Bacterial Isolates Associated with Algal Attachment

**Fig. 3:**
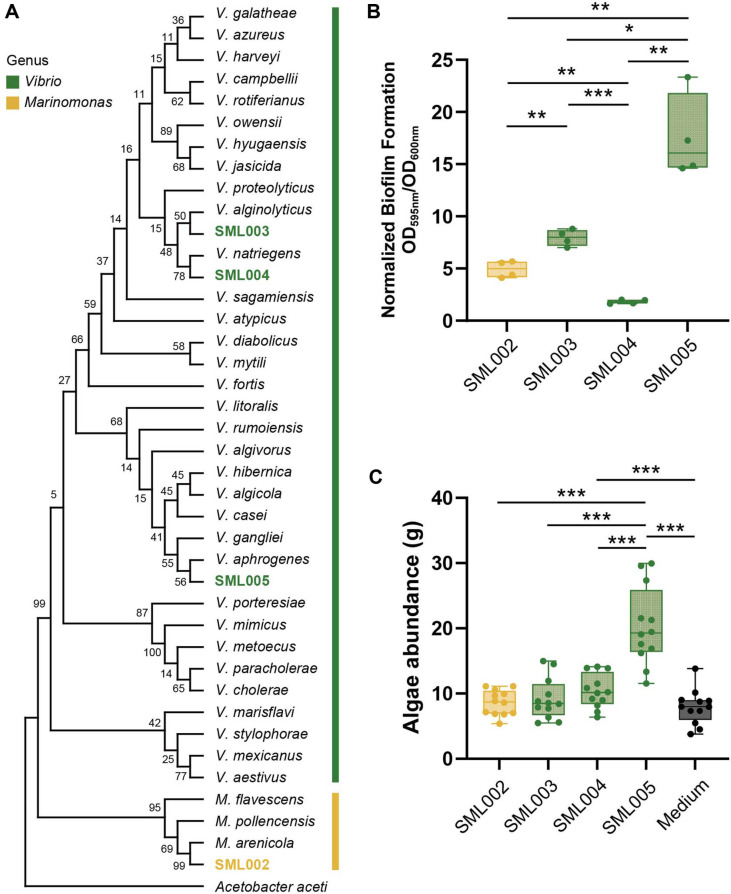
Phylogenetic characterization and functional traits of bacterial isolates from brown algae. (**A**) Maximum-likelihood phylogenetic tree based on near full-length 16S rRNA gene sequences of bacterial isolates obtained from brown algae. The tree was inferred using the Jukes–Cantor model with gamma-distributed rates, and node support was assessed by 1,000 bootstrap replicates. Gaps were treated by partial deletion (95% site coverage). (**B**) Quantification of biofilm formation ability of bacterial isolates. Biofilm production was measured using 0.1% crystal violet staining and optical density-based quantification after dye solubilization. Values were normalized to the culture medium control and are presented as box plots representing biological replicates. (**C**) Effects of bacterial isolates on algal biomass assessed by co-culturing with *U. pinnatifida* female gametophytes. Algal biomass was quantified by qPCR targeting the housekeeping gene eEF1β, and absolute quantification was performed using a standard curve. Uninoculated medium served as a blank control. Statistical significance was assessed using Student’s t-test (***, *p* < 0.001; **, *p* < 0.01; *, *p* < 0.05).

We aimed to bridge the gap between community-level observations and functional verification by isolating key symbiotic bacteria representing the attached and drifting states. Specifically, we prioritized the isolation of *Vibrio* species as the primary functional candidates for attachment, while *Marinomonas* was selected as a negative control because it was identified as a representative genus enriched in drifting samples, contrasting with the attachment-enriched distribution of *Vibrio* (Fig. 2A and 2B). Through extensive cultivation using both general Marine agar and selective TCBS agar, a total of three *Vibrio* strains and one *Marinomonas* strain were successfully obtained. Phylogenetic analysis based on 16S rRNA gene sequences confirmed their taxonomic identities, showing clear affiliations with their respective lineages (Fig. 3A and S3). Among them, SML003 is closely related to *V. natriegens* clade, a species renowned for its exceptionally rapid growth rate [45], while SML004 is associated with *V. alginolyticus* clade, a taxon known for its high pathogenicity and versatile colonization across diverse marine organisms [46-49]. Furthermore, strain SML005 was found to be closely related to the *V. aphrogenes* clade, a group typically found in macroalgal-associated environments [50]. Strain SML002 was identified as *M. arenicola* clade, a species originally reported to be isolated from marine sediments [51]. These isolates provide the essential biological material required to experimentally validate the causal roles of specific microbial communities associated with different attachment states.

As *Vibrio* species are known to form biofilms [52], and biofilms have been linked to microbial communication and host interactions [26], the biofilm-forming ability of the isolated strains was evaluated (Fig. 3B). Among the *Vibrio* strains, SML005 exhibited substantially higher biofilm formation (17.5 ± 4.1) compared to the others (SML003, 1.8 ± 0.2; SML004, 8.0 ± 0.8) with significant differences observed across pairwise comparisons (*p* < 0.05). In contrast, SML002 showed relatively low biofilm formation (4.9 ± 0.8), indicating clear differences in surface colonization potential between taxa.

To assess whether pre-formed bacterial biofilms could promote brown algal settlement and attachment, co-culture experiments were conducted using *U. pinnatifida* female gametophytes (Fig. 3C). The strain exhibiting the highest biofilm formation (*V. aphrogenes* clade SML005) also resulted in the most significant increase in attached algal biomass. Specifically, SML005-treated groups reached a mean abundance of 20.3 ± 6.0, representing an approximately 2.57-fold increase compared to the medium control (7.9 ± 2.7), which was statistically significant (*p* < 0.05). In contrast, the other *Vibrio* strains (SML003: 10.4 ± 2.6; SML004: 9.2 ± 3.2) and the *Marinomonas* isolate (SML002: 8.6 ± 1.9) showed abundance levels comparable to the control, suggesting that the promotion of algal development is a strain-specific trait rather than a general characteristic of the genus.

## Discussion

In this study, we comprehensively characterized brown algal-associated microbiomes across diverse coastal regions and identified attachment status as a key factor structuring microbial community composition and function. Our results revealed that geographic location significantly structures microbial diversity, with the West Sea forming a distinct cluster separate from all other coastal regions. This separation likely reflects the unique environmental conditions of the West Sea, which, unlike other coastal areas, is characterized by higher turbidity and fluctuating salinity [53]. Despite these regional differences, the consistent shifts in community structure across all sites underscore that the host's physiological state, particularly its attachment status, exerts a more dominant impact than regional environmental variations. We included the holdfast as the tissue involved in substrate attachment [54] to evaluate host–microbe interactions at the attachment interface. We demonstrate that specific bacterial taxa are differentially associated with attached and drift algal states and contribute to distinct functional potentials by integrating amplicon sequencing, Random Forest-based feature selection, and cultivation-based validation.

Our study showed that *Vibrio* was consistently identified as a key taxon associated with attached brown algal samples, and co-culture experiments further demonstrated that the specific *V. aphrogenes* clade strain SML005 can enhance algal abundance. These findings suggest that *Vibrio* may play an active role in interactions with brown algae. *Vibrio* species are more frequently detected on algal surfaces [55, 56] or sediments [57, 58] than in free-living states in the water column. This pattern is likely linked to biofilm formation on both biotic and abiotic surfaces, which facilitates long-term persistence and efficient colonization [59, 60].

Microbial biofilms are complex systems characterized by the secretion of extracellular polymeric substances (EPS), which create a robust matrix that stabilizes the microbial community [61]. The settlement of zoospores onto suitable substrates is a critical step in the life cycle of many macroalgae. Some zoospores selectively attach to surfaces harboring bacterial biofilms [15, 25, 62, 63], but their recruitment is markedly reduced in the absence of EPS [64]. This demonstrates that the EPS matrix itself can promote settlement. While zoospores are responsible for initial settlement, the transition through germination and development into gametophytes represents a critical step for stable establishment and persistence on the substrate [65]. In our study, *V. aphrogenes* clade strain SML005 was the only isolate that showed both high biofilm formation and a significant increase in algal biomass. It resulted in a 2.57-fold increase during co-culture experiments. These results strongly suggest that the biofilm from *V. aphrogenes* clade strain SML005 provides a specialized functional surface that facilitates the settlement of *U. pinnatifida* female gametophytes.

Bacteria perform quorum-sensing signaling by releasing acyl-homoserine lactones (AHLs) to initiate biofilm formation [61]. Research has shown that AHLs regulate spore release in several algal taxa, including Ulvaceae [64, 66], *Gracilaria* [67], and *Acrochaetium* [28]. Close relatives of our isolates (SML003, SML004, and SML005) have been reported to produce AHLs [68-70]. Although AHL production was not directly measured in this study, the strong biofilm-forming capacities observed in the isolated *Vibrio* strains, together with previous reports of AHL production within the *Vibrio* genus [68-70], suggest that quorum-sensing-mediated biofilm formation may contribute to early interactions between these bacterial strains and their brown algal hosts. Although these mechanisms were not directly examined in the present study, they represent plausible pathways through which SML005 may facilitate early brown algal settlement and warrant further investigation.

PICRUSt2-based predictions indicated that *Vibrio*-associated functional modules, including nicotinate degradation, suggest the utilization of vitamin-like compounds within the phycosphere. Algae have been reported to be a rich source of vitamins, including nicotinate and other B vitamins, indicating that algal biomass may provide accessible organic compounds in marine environments [71, 72]. This availability of nutrients may facilitate *Vibrio* colonization on macroalgal surfaces by supporting metabolic activity. Colonized *Vibrio* may contribute to host-associated processes through previously reported beneficial interactions in marine systems, including reduced bleaching incidence in macroalgae [73] and metabolic exchanges with symbiotic algae such as nutrient cycling and vitamin-related interactions [74]. However, given the well-documented pathogenic potential of *Vibrio* [75], these findings should be interpreted with caution. Generally, *Vibrio* bacteria maintain a beneficial or neutral symbiotic relationship, but environmental factors such as temperature or cell density can turn *Vibrio* bacteria pathogenic [76, 77]. While the potential for pathogenicity under stress remains a concern, their high abundance in attached samples and diverse metabolic capabilities suggest that some lineages may offer ecological benefits to the host.

The isolated *M. arenicola* clade strain (SML002) exhibited relatively low biofilm formation and showed no significant effect on algal attachment or growth in co-culture experiments compared to the control. Members of the genus *Marinomonas* have frequently been reported as core components of macroalgal-associated microbial communities, indicating a stable association with marine primary producers [78-80]. This genus is known to metabolize algal-derived compounds such as DMSP, one of the most abundant organosulfur compounds in marine environments that plays a key role in global sulfur and carbon cycling [10, 81]. Such metabolic capabilities suggest an ecological strategy centered on the utilization of dissolved organic matter released by macroalgae. Consistent with this interpretation, the enrichment of *Marinomonas* in drifting algal samples observed in this study may reflect adaptation to nutrient acquisition from degradation-derived organic substrates in the surrounding microenvironment.

In this study, we showed that brown algal-associated microbial communities differ according to attachment status, with consistent taxonomic and functional shifts between attached and drifting samples. These differences appear to be driven by distinct ecological strategies, with *Vibrio* associated with host interaction and *Marinomonas* linked to environmental resource utilization within the phycosphere, the immediate microscopic region surrounding algal cells. Although the *V. aphrogenes* clade strain SML005 was found to promote algal settlement, the underlying genetic and molecular mechanisms remain unresolved. Further studies are needed to clarify the causal basis of this interaction. Furthermore, our co-culture experiments were conducted under controlled laboratory conditions, which may differ from complex natural environments. Large-scale field validation is necessary to confirm the stability of these symbiotic relationships in natural ecosystems. Expanding the host range by inoculating gametophytes from other brown algae or red algae would also demonstrate the versatility of this candidate symbiont. Overall, this study highlights the importance of host attachment status and microbial functional potential in understanding algal microbiome organization.

## Supplemental Materials

Supplementary data for this paper are available on-line only at http://jmb.or.kr.
